# Dynamics of tandemly repeated DNA sequences during evolution of diploid and tetraploid botiid loaches (Teleostei: Cobitoidea: Botiidae)

**DOI:** 10.1371/journal.pone.0195054

**Published:** 2018-03-28

**Authors:** Alexandr Sember, Jörg Bohlen, Vendula Šlechtová, Marie Altmanová, Šárka Pelikánová, Petr Ráb

**Affiliations:** 1 Laboratory of Fish Genetics, Institute of Animal Physiology and Genetics, Czech Academy of Sciences, Rumburská 89, Liběchov, Czech Republic; 2 Department of Ecology, Faculty of Science, Charles University, Viničná 7, Prague 2, Czech Republic; University of Florence, ITALY

## Abstract

Polyploidization has played an important role in the evolution of vertebrates, particularly at the base of Teleostei–an enormously successful ray-finned fish group with additional genome doublings on lower taxonomic levels. The investigation of post-polyploid genome dynamics might provide important clues about the evolution and ecology of respective species and can help to decipher the role of polyploidy *per se* on speciation. Few studies have attempted to investigate the dynamics of repetitive DNA sequences in the post-polyploid genome using molecular cytogenetic tools in fishes, though recent efforts demonstrated their usefulness. The demonstrably monophyletic freshwater loach family Botiidae, branching to evolutionary diploid and tetraploid lineages separated >25 Mya, offers a suited model group for comparing the long-term repetitive DNA evolution. For this, we integrated phylogenetic analyses with cytogenetical survey involving Giemsa- and Chromomycin A_3_ (CMA_3_)/DAPI stainings and fluorescence *in situ* hybridization with 5S/45S rDNA, U2 snDNA and telomeric probes in representative sample of 12 botiid species.

The karyotypes of all diploids were composed of 2n = 50 chromosomes, while majority of tetraploids had 2n = 4x = 100, with only subtle interspecific karyotype differences. The exceptional karyotype of *Botia dario* (2n = 4x = 96) suggested centric fusions behind the 2n reduction. Variable patterns of FISH signals revealed cases of intraspecific polymorphisms, rDNA amplification, variable degree of correspondence with CMA_3_^+^ sites and almost no phylogenetic signal. In tetraploids, either additivity or loci gain/loss was recorded. Despite absence of classical interstitial telomeric sites, large blocks of interspersed rDNA/telomeric regions were found in diploids only.

We uncovered different molecular drives of studied repetitive DNA classes within botiid genomes as well as the advanced stage of the re-diploidization process in tetraploids. Our results may contribute to link genomic approach with molecular cytogenetic analyses in addressing the origin and mechanism of this polyploidization event.

## Introduction

Polyploidization events have played an important role since the early evolution of vertebrates, with a spectacular example of teleostean fishes, where this entire lineage experienced the so-called teleost-specific whole genome duplication (TS-WGD) approximately between 226–316 million years ago (Mya) [1], following their divergence from the rest of actinopterygian fishes. TS-WGD had undoubtedly a major impact on the processes shaping the teleost genomes and to spur the genome innovations [2,3], while its causative link with diversification and evolutionary success of this fish clade remains yet controversial [4–6]. Besides, additional whole-genome duplications (WGDs) took place independently in several teleostean lineages–e.g., Catostomidae [7,8], Cyprinidae [9–13], Cobitidae (e.g., [14]), Callichthyidae [15] and Salmoniformes [16].

Polyploidization is usually accompanied by large-scale and genome-wide changes that are extensively complex and include–among others–DNA sequence loss, various chromosome rearrangements, changes in gene expression and epigenetic modifications. These processes are acting in a species-specific manner, leading to distinct signs and various extent of post-WGD genome restructuring in order to restore the diploid-like inheritance, to buffer the parental genomes' incompatibilities and/or to prevent meiotic irregularities (e.g., [17–22]. As an integral part of these processes, rapid changes in the amount and composition of repetitive DNA content occur both on the level of immediate and long-term post-polyploid genome evolution [19–24]. Distinct repetitive DNA sequences may undergo either biased elimination, leading in vast majority of cases to the so-called genome downsizing; or they can be amplified and/or accumulated in gene-poor regions. These changes are thought to be driven by ectopic (non-allelic) recombination, greatly enhanced by deregulated control of (retro-) transposition activity [20,23–26].

All these facts imply that cytogenetic mapping of repetitive DNA classes might provide useful tool for elucidating the dynamics of post-polyploid genomes. In fishes, several attempts have been conducted rather in nascent or synthetic polyploids (e.g., [27–33]) and in genetically manipulated fishes [34]. Nonetheless, also old-aged WGDs have been recently examined with success, bringing novel important insights into several long-standing issues [35–38].

The freshwater fish family Botiidae represents one of the 10 major lineages within the cypriniform superfamily Cobitoidea [39]. Botiids comprise eight genera with 58 recognized species [40], with wide distribution throughout South-, East- and Southeast Asia. Many species are attractive for ornamental fish trade due to conspicuous colour variations. Phylogenetic reconstructions [41–43] showed that the family contains two main evolutionary lineages–the subfamilies Leptobotiinae and Botiinae–that are long-time separated from each other. Bearing in mind that all species from the subfamily Leptobotiinae are primarily diploid, whereas those in Botiinae are (paleo-) tetraploids [42,44], we can assume that the separation of both sub-lineages stems from a single evolutionary event, accompanied or directly driven by the whole genome duplication [42]. Although until now the split of these two sublineages has not been precisely dated, it is reasonable to assume that since Botiinae contains all species on the Indian subcontinent, and this region has been isolated from the rest of Asia by the uplift of the Himalayan Mountain since 28 Mya [45, 46], the origin of the polyploid subfamily must have taken place before this event.

To date, cytogenetic reports in Botiidae are limited to conventional Giemsa-stained karyotypes or basic chromosome counts [42,44,47–49], while molecular cytogenetic data like those scarcely published in sister loach groups (namely Cobitidae and Nemacheilidae; [50–55]) are yet non-existent for this lineage.

Containing two sister lineages with diploid and tetraploid levels, botiid loaches represent a suited model to study the differential patterns of diploid vs. post-polyploid genome evolution at the chromosomal level and to track the long-term repetitive DNA dynamics using molecular cytogenetic approach. Hence, we utilized conventional and molecular cytogenetic protocols, with the latter involving chromosome mapping of 5S and 45S rDNA and U2 snDNA sites and telomeric (TTAGGG)_*n*_ repeats through FISH analysis and we interpreted the observed patterns within a phylogenetic framework. Our results uncovered different molecular drives of selected cytogenetic markers and the data collectively point to the advanced stage of genome re-diploidization, with mosaic of diploid and tetraploid genomic regions in all studied tetraploids, corroborating the view of old-aged and rather single WGD event that predated Leptobotiinae/Botiinae divergence.

## Materials and methods

### Ethical statement

The experimental procedures with fishes were approved by the Institutional Animal Care and Use Committee of the IAPG AS CR, according to directives of the State Veterinary Administration of the Czech Republic, permit number 217/2010, and by permit number CZ 02386 from the Ministry of Agriculture of the Czech Republic. All surgery was performed under phenoxyethanol anesthesia, and all efforts were made to minimize suffering.

### Animals

**Fig 1:**
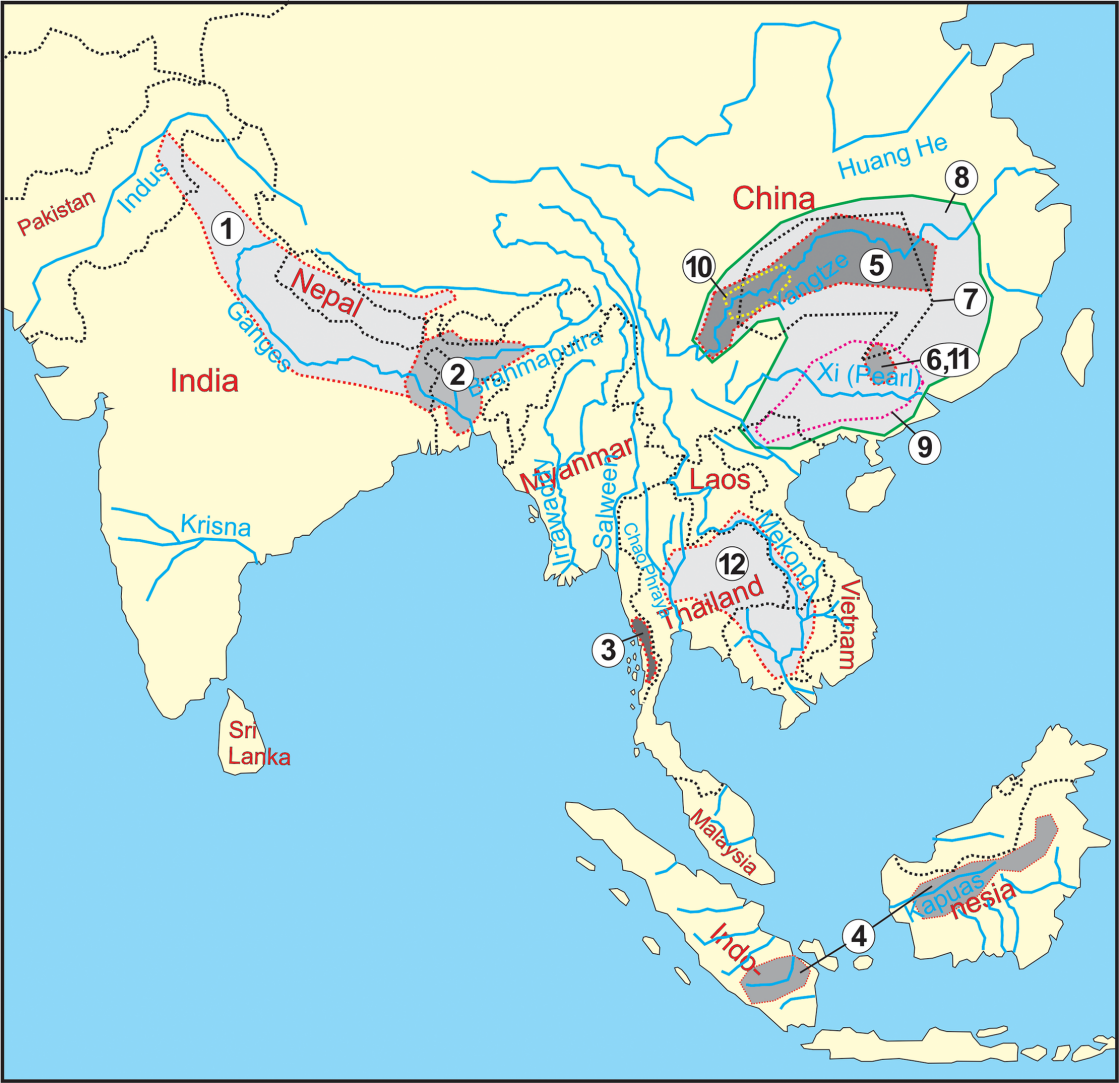
Distribution areas of the investigated species of Botiidae. 1 –*B*. *almorhae*, 2 –*B*. *dario*, 3 –*B*. *udomritthiruji*, 4 –*Ch*. *macracanthus*, 5 –*L*. *elongata*, 6 –*L*. *guilinensis*, 7 –*L*. *microphthalma*, 8 –*P*. *fasciatus*, 9 –*S*. *pulchra*, 10 –*S*. *superciliaris*, 11 –*S*. *zebra*, 12 –*Y*. *lecontei*.

In this study, 31 individuals representing 12 species (four diploid and eight tetraploid) of the Botiidae family were analyzed (Table 1). The distribution areas of the examined species are shown in Fig 1. All analyzed individuals were obtained from the ornamental fish trade and identified by a trained loach taxonomist (JB). Voucher specimens were deposited to the Fish collection of the Laboratory of Fish Genetics, IAPG, CAS, Liběchov.

**Table 1:** List of analyzed species of Botiidae, sample sizes and distribution areas.

Subfamily	Species	N	ID codes	Type locality
Botiinae	*Botia almorhae* (Gray, 1831)	3	A0425, A0426, A10048	Uttarakhand: Almorah (India)
(4n)	*Botia dario* (Hamilton, 1822)	1	A7553	India and Bangladesh
	*Botia udomritthiruji* (Ng, 2007)	2	A4830, A4831	Taninthayi: Attaran River (Myanmar)
	*Chromobotia macracanthus* (Bleeker, 1852)	3	A9180, A9181, A9826	Sumatra: Kwanten River (Indonesia)
	*Sinibotia pulchra* (Wu, 1939)	3	A5286, A5287, A3682	Guangxi: Li-Kiang at Yangso (China)
	*Sinibotia superciliaris* (Günther, 1892)	3	A8527, A8528, A8529	Sichuan: Kia-tiang-fu, foot of Omie-shan (China)
	*Sinibotia zebra* (Wu, 1939)	3	A5277, A5279, A5280	Guangxi: Li-Kiang at Yangso (China)
	*Yasuhikotakia lecontei* (Fowler, 1937)	2	A10725, A10727	Ubon Ratchathani: Khemarat (Thailand)
Leptobotiinae	*Leptobotia elongata* (Bleeker, 1870)	2	A8392, A8393	Hunan: Dongting Lake (China)
(2n)	*Leptobotia guilinensis* (Chen, 1980)	4	A5267, A5270, A5272, A5273	Guangxi: Li-Kiang at Yangso (China)
	*Leptobotia microphthalma* (Fu and Ye, 1983)	3	A5294, A5285	Sichuan: Leshan County: Min River drainage (China)
	*Parabotia fasciatus* (Dabry de Thiersant, 1872)	1♂, 1♀	A8391 A3680	Yangtze River (China)

### Chromosome preparations

Mitotic chromosomes were obtained mostly from regenerating fin tissue as described by Völker and Ráb [56], with the modifications of Sember et al. [53] and altered times of fin regeneration (ranging from three to six weeks). In several cases, chromosome preparations from head kidney [57] and/or from the lymphocyte cultures [58,59] were performed. Initially, chromosomes were stained with 5% Giemsa solution (pH 6.8) (Merck, Darmstadt, Germany) for basic cytogenetic analysis. Selected slides were then destained in cold fixation with methanol: acetic acid 3:1 (v/v) and re-used for the other techniques. For fluorescence *in situ* hybridization (FISH), slides were dehydrated in an ethanol series (70%, 80% and 96%, 3 min each) and stored in a freezer (-20°C).

At least ten metaphases per specimen and method were analyzed, mostly sequentially. In a few metaphases, plates with incomplete 2n were selected to demonstrate patterns of a particular marker (see the figure legends). Chromosomes were classified according to Levan et al. [60], but modified as m–metacentric, sm–submetacentric, st–subtelocentric, a–acrocentric, where st and a chromosomes were scored as uni-armed to calculate NF value (Nombre Fondamental, number of chromosome arms).

### CMA_3_/DAPI staining

Fluorescent staining was performed sequentially or in separate experiments by GC-specific fluorochrome Chromomycin A_3_ (CMA_3_) (Sigma-Aldrich) and AT-specific fluorochrome DAPI (Sigma-Aldrich), following Mayr et al. [61] and Sola et al. [62].

### DNA isolation and probe preparation

Whole genomic DNA was extracted from fin tissue using the i) conventional phenol-chloroform-isoamylalcohol method [63] with PhaseLock Eppendorf tubes (5PRIME, Gaithersburg, USA) to prevent protein contamination, or ii) the Qiagen DNAeasy Blood & Tissue Kit (Qiagen, Hilden, Germany). 5S and 28S rDNA fragments were obtained by polymerase chain reaction (PCR) using primers and thermal profiles described in Sember et al. [53]. U2 snDNA amplification was done by PCR with primers: U2F (5’-ATCGCTTCTCGGCCTTATG-3’) and U2R (5’-TCCCGGCGGTACTGCAATA-3’) [64], using thermal profile described in Scacchetti et al. [65]. The resulting PCR products were purified using NucleoSpin Gel and PCR Clean-up (Macherey-Nagel GmbH, Düren, Germany). DNA fragments of U2 snDNA were cloned to pDrive Cloning Vector (Qiagen) and transformed into QIAGEN EZ Competent Cells (Qiagen). Selected recombinant plasmids were isolated by QIAprep Spin Miniprep Kit (Qiagen) and sequenced in both strands by using BigDye™ Terminator Cycle Sequencing Kit v.1.1 (PE Applied Biosystems, Darmstadt, Germany) according to manufacturer's instructions and products purified with DyeEx Spin Kit (Qiagen). Sequencing was performed on ABI Prism 3130 (Applied Biosystems). Chromatograms of obtained sequences were assembled using SeqMan Pro 10.1.2 (LaserGene, DNASTAR, Madison, Wl). The sequences were aligned and manually revised in BioEdit 7.0.5.3 [66]. The resulting consensus sequences were confirmed using NCBI BLAST/N analysis [67] and selected clones used to construct FISH probes. For construction of 5S and 28S rDNA probes, cloned fragments from the botiid species *Botia almorhae* (5S and 28S) and the nemacheilid species *Schistura bolavenensis* (28S) were utilized (for details, see Sember et al. [53]).

DNA probes were labelled in a PCR reaction with biotin-16-dUTP (Roche, Mannheim, Germany) or digoxigenin-11-dUTP (Roche), respectively. For each slide, 200 ng of one (uni-colour FISH) or two (dual-colour FISH) probes and 25 μg of sonicated salmon sperm DNA (Sigma-Aldrich) were used. The final hybridization mixtures were prepared according to Sember et al. [53].

Telomeric (TTAGGG)_*n*_ repeats were detected by FISH using a commercial telomere PNA (peptide nucleic acid) probe directly labelled with Cy3 (DAKO, Glostrup, Denmark) according to the manufacturer's instructions, with a single modification concerning the prolonged hybridization time (1.5 h).

### FISH analysis

Dual-colour FISH experiments were performed essentially according to Sember et al. [53]. Probes were detected by Anti-Digoxigenin-FITC (Roche) and Streptavidin-Cy3 (Invitrogen Life Technologies, San Diego, CA, USA). Experiments with altered labelling (e.g., biotin for 28S and digoxigenin for 5S rDNA) were included to verify the observed patterns. All FISH images presented here have pseudocoloured signals–red for the 28S rDNA and U2 snDNA probes and green for the 5S rDNA. In uni-colour FISH, hybridization signals of U2 snDNA probes were detected using the Cy3-conjugated streptavidin (Invitrogen, San Diego, CA, USA), followed by additional signal enhancement using biotinylated Anti-Streptavidin and second round of Streptavidin-Cy3 detection (Vector Laboratories, Burlingame, USA) according to Fuková et al. [68]. Finally, all FISH slides were mounted in medium containing antifade and 1.5 μg/ml DAPI (Cambio, Cambridge, United Kingdom).

### Microscopy and image processing

Giemsa-stained chromosomes and FISH images were inspected using a Provis AX70 Olympus microscope with a standard fluorescence filter set. FISH images were captured under immersion objective 100× with a black and white CCD camera (DP30W Olympus) for each fluorescent dye using Olympus Acquisition Software. The digital images were then pseudocoloured (blue for DAPI, red for Cy3, green for FITC) and superimposed with MicroImage software (Olympus, version 4.0). Karyotypes from Giemsa-stained chromosomes were arranged in IKAROS (Metasystems) software. Final images were optimized and arranged using Adobe Photoshop, version CS6.

### Molecular phylogenetic analyses

The phylogenetic hypothesis was based on the analyses of three molecular markers: mitochondrial cytochrome *b* (*cyt b*) gene and nuclear recombination-activating gene 1 (*RAG1*) and interphotoreceptor retinoid-binding protein (*IRBP*). The primers and PCR reaction protocols for *cyt b* and *RAG1* followed Šlechtová et al. [42] and Šlechtová et al. [69], and Chen et al. [70] for the *IRBP* amplification. All three genes were sequenced for each of the 39 analyzed specimens of Botiidae.

Chromatograms were edited and assembled using SeqMan Pro 10.1.2 (LaserGene, DNASTAR). The sequences were aligned in BioEdit 7.0.5.3 [66] and evaluated based on their amino acid translation.

Alignments of all three genes were concatenated into a single 3020 bp dataset (1116 bp of *cyt b*, 910 bp of *RAG1* and 994 bp of *IRBP*). The sequences with GenBank accession numbers KU517025-KU517132 were published in Bohlen et al. [49]. Newly obtained sequences were deposited in GenBank under the accession numbers MF681728 to MF681780.

The phylogenetic analysis of the concatenated dataset was performed using the partitioned Bayesian inference in MrBayes 3.2.2 [71]. The dataset was partitioned by genes and codon positions, involving in total nine partitions. Prior to the analyses, the MEGA 5.10 software [72] was used to estimate the most suited model for each gene partition under the Bayesian information criterion (BIC). The Bayesian analyses were performed in two independent runs of 10 million generations, each employing six Markov chain Monte Carlo (MCMC) analyses, with default heating conditions, starting with random trees and a sampling frequency of each 100 generations. The parameter settings corresponded to the best-fit models. After applying a burn-in of first 25% of generated trees, a 50% majority rule consensus tree was built and statistical supports of clades were assessed by posterior probabilities.

## Results

### Sequence analysis of *RAG1*, *IRBP* and *cyt b*

The phylogenetic relationships between the analyzed specimens were reconstructed using the mitochondrial cytochrome *b* gene and the nuclear genes *RAG1* and *IRBP*. All acquired reconstructions were highly congruent, independent of the gene used. Consequently, the datasets were concatenated into a single matrix of 3020 bp.

**Fig 2:**
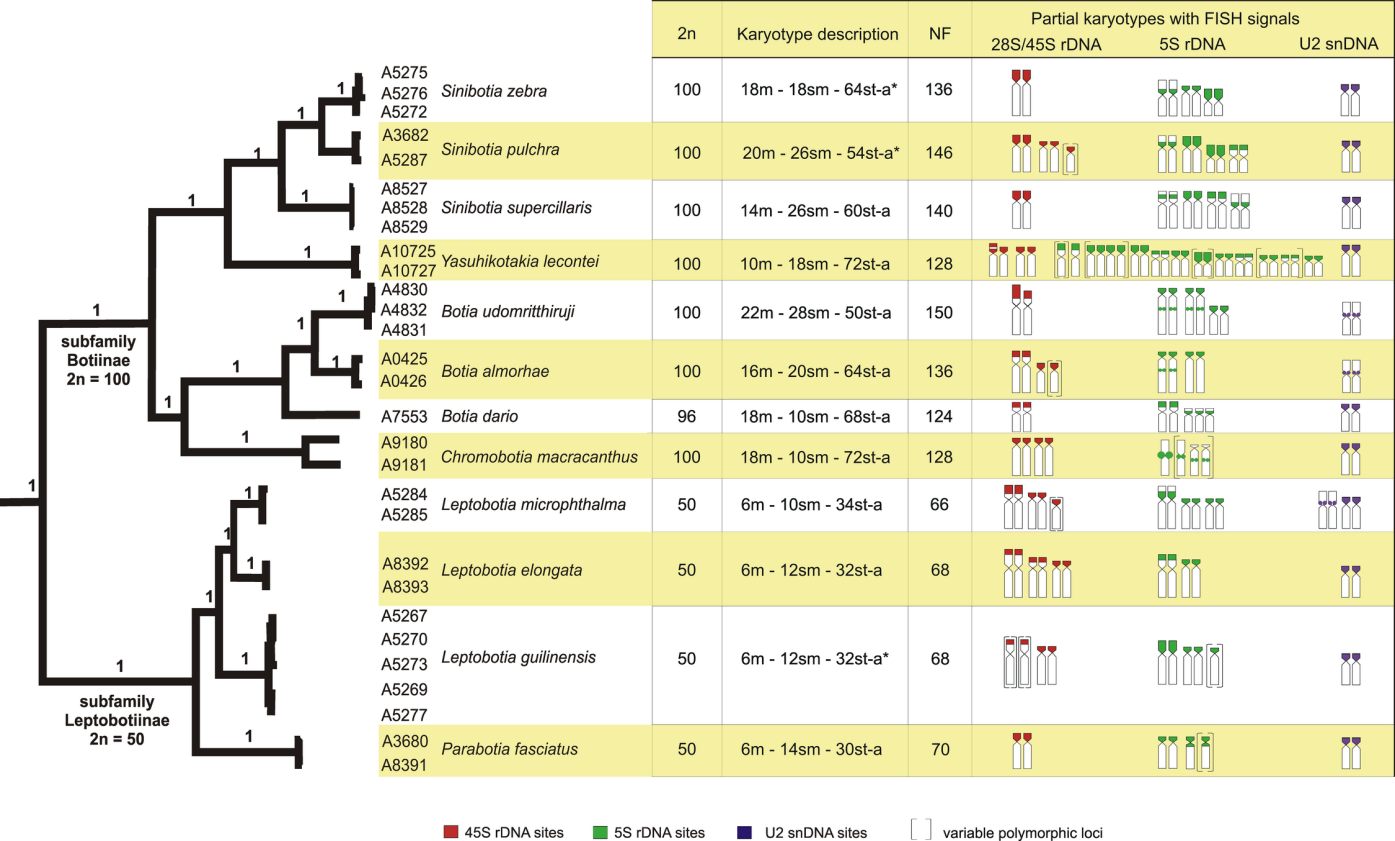
Phylogenetic relationships and karyotype characteristics of inspected botiids. 2n, karyotype description, NF and idiograms showing chromosomes bearing 45S (red), 5S (green) rDNA and U2 snDNA (violet) sites are plotted onto phylogenetic tree obtained by Bayesian analysis based on the mitochondrial (*cyt b*) and nuclear (*RAG1*, *IRBP*) genes. Polymorphic rDNA sites are in brackets. The asterisk denotes species' karyotypes already published in Bohlen et al. [49], with the st and a chromosome pairs being here scored together in one st-a category.

The resulting phylogenetic analysis of the concatenated dataset identified two major lineages; the one collecting all four species of the subfamily Leptobotiinae and the other all eight species of the subfamily Botiinae (Fig 2). Within the Leptobotiinae, the single species of *Parabotia* was sister to a group collecting the three analyzed species of *Leptobotia*. Within the Botiinae, two sublineages were visible, one containing the single species of *Chromobotia* plus a cluster of three species of *Botia*, while the second sublineage was composed from the single species of *Yasuhikotakia* as sister to a group of three species of *Sinibotia*.

All recovered lineages had the maximum statistical support in all analyses. In addition, all analyzed species were identified as monophyletic and well-separated lineages with high statistical support.

### Sequence analysis of U2 snDNA

PCR amplification of U2 snDNA resulted consistently in a fragment approximately 180 bp in size, containing partial sequence of U2 snRNA coding region. Searches with the BLAST/N program at NCBI yielded repeatedly high similarity results with the U2 snRNA gene region of, e.g., the perciform fish *Argyrosomus regius* (95% identity; e.g., GenBank accession number JF799429.1) or platyfish *Xiphophorus maculatus* (95% identity; e.g., GenBank accession number XR_002753210.1). Sequences for six species (diploid *L*. *elongata* and *P*. *fasciatus* and tetraploid *B*. *almorhae*, *Ch*. *macracanthus*, *S*. *pulchra* and *Y*. *morleti–*with the latter representing another botiid species, not subjected to cytogenetic/phylogenetic analyses in this study) were deposited in GenBank (accession numbers MG874999-MG875004).

### Patterns of karyotype differentiation

Fig 2 provides an overview about 2n, karyotype composition, NF and chromosomal distribution of 5S, 45S rDNA and U2 snDNA sites mapped onto a phylogenetic framework. The present study gives the first karyotype description of *B*. *almorhae*, *B*. *udomritthiruji* and *L*. *microphthalma*, while karyotypes of further six species were described formerly and are revised here. The remaining species under study, namely *L*. *guilinensis*, *S*. *pulchra* and *S*. *zebra*, had been karyologically examined in our previous work [49]. In addition, the present study extended for the first time the basic karyology in this fish group by mapping of tandemly repeated DNA sequences in all species under study.

#### Karyotype analysis

Karyotypes of all analyzed species were composed of comparatively small chromosomes, gradually decreasing in size, which made the estimation of karyotype composition difficult. All karyotypes displayed slight prevalence of uni-armed elements. Chromosome complements of the tetraploids showed apparently smaller overall size compared to diploids.

**Fig 3:**
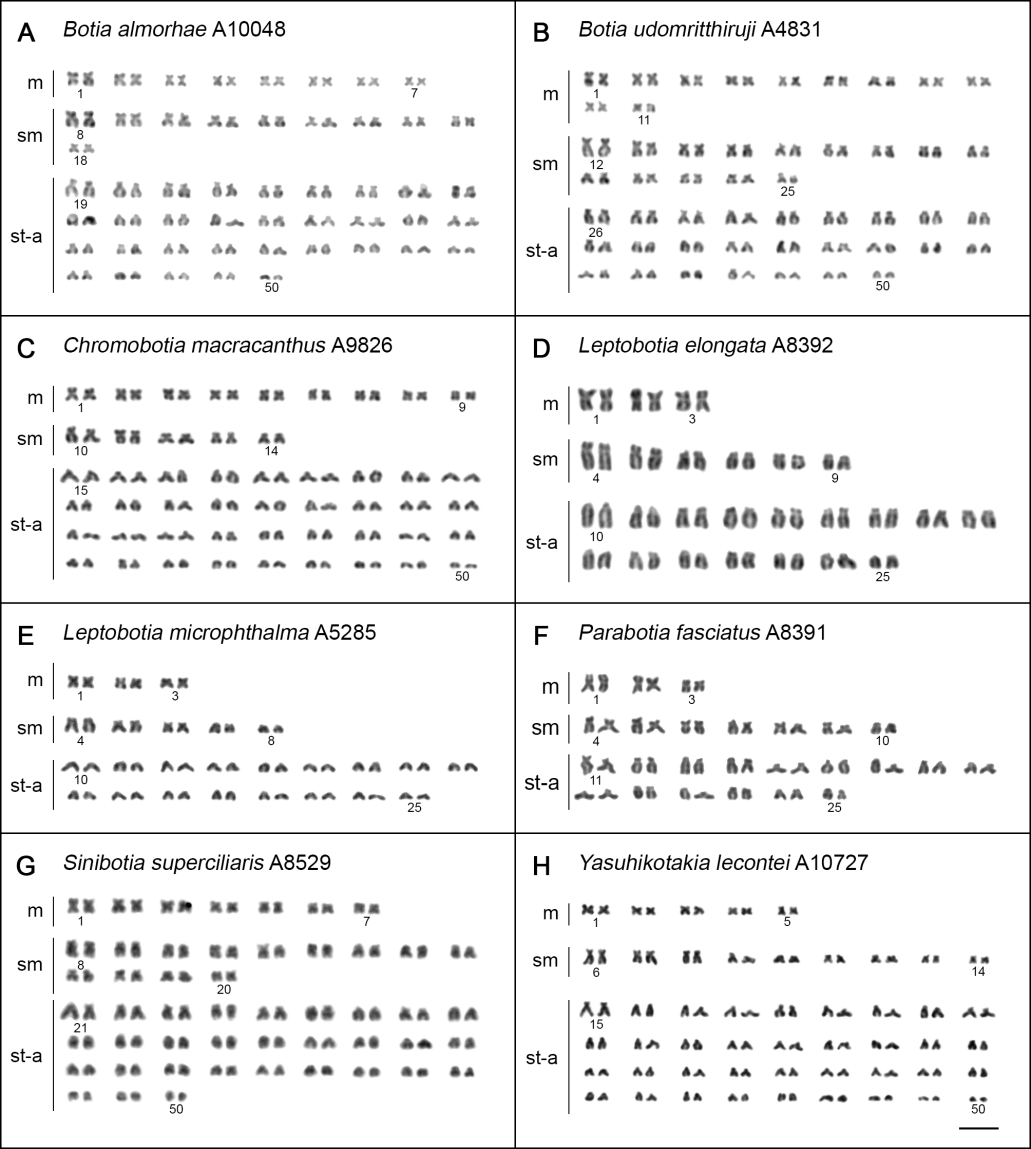
Karyotypes of botiid species after Giemsa staining. (**A**) *B*. *almorhae*, (**B**) *B*. *udomritthiruji*, (**C**) *Ch*. *macracanthus*, (**D**) *L*. *elongata*, (**E**) *L*. *microphthalma*, (**F**) *P*. *fasciatus*, (**G**) *S*. *superciliaris*, (**H**) *Y*. *lecontei*. Bar = 10 μm.

In all but one species the chromosome number corresponded to their diploid (2n = 2x = 50) and tetraploid (2n = 4x = 100) level, respectively (Figs 2 and 3). A single tetraploid species *B*. *dario* displayed karyotype with reduced 2n = 96, with presence of four distinctly large metacentric chromosomes (S1A Fig). As preparations from a single specimen of *B*. *dario* in our sampling provided only a limited number of complete and/or well-spread metaphases, the results obtained are rather treated with caution and presented in separate file (S1 Fig; for details, see a figure legend).

A notably conservative karyotype structure was observed among the diploid botiid species under study, with only minor differences in karyotypes and NF values ranging from 66 to 70 (Figs 2 and 3). while species of tetraploid origin showed slightly more variability in this sense (Fig 3, S1A Fig), with NF ranging from 124 to 150 (Fig 2). No intraspecific karyotype variability was evidenced within our sampling.

#### CMA_3_/DAPI staining

**Fig 4:**
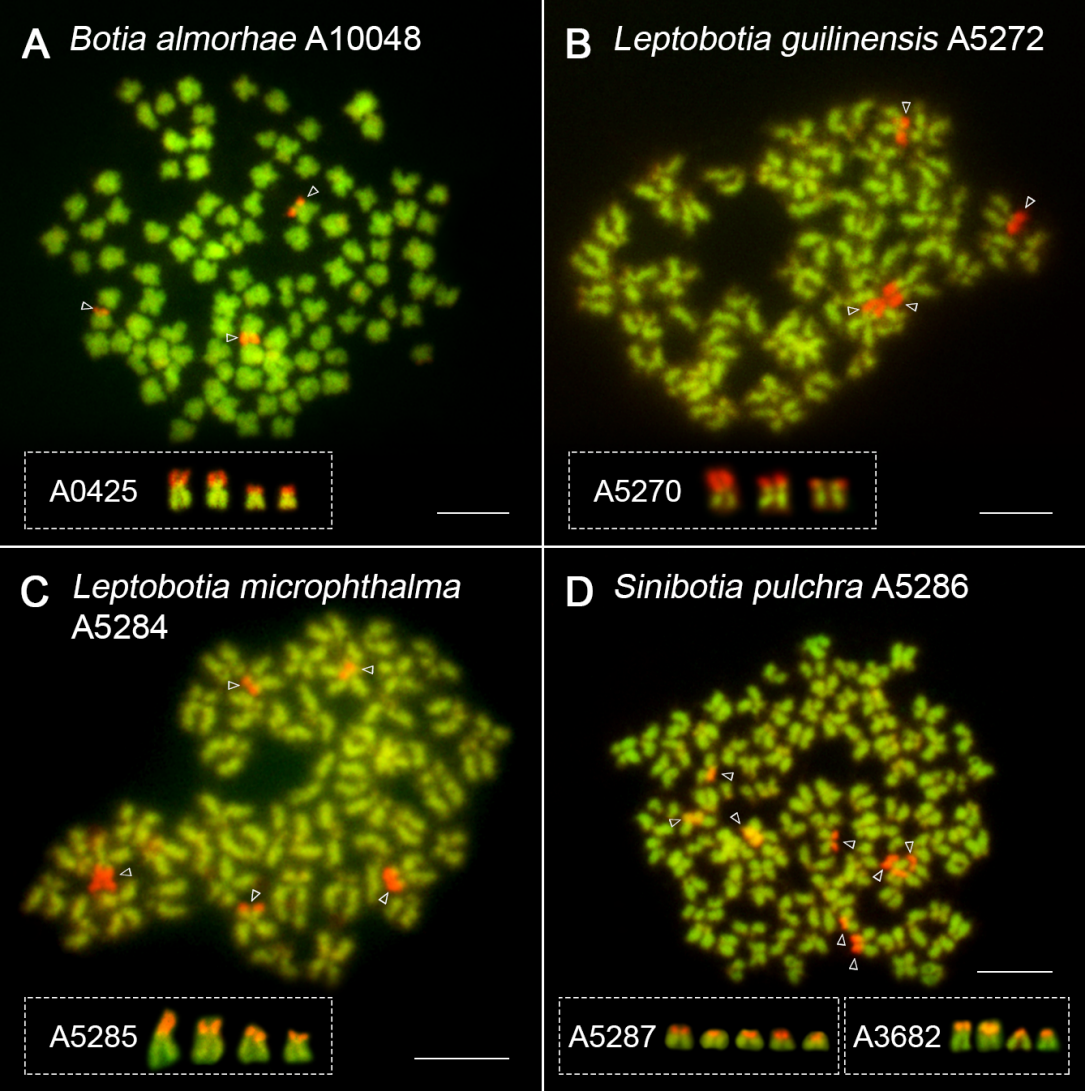
CMA_3_/DAPI staining in selected botiid species. Mitotic metaphases of (**A**) *B*. *almorhae*, (**B**) *L*. *guilinensis*, (**C**) *L*. *microphthalma*, (**D**) *S*. *pulchra*. For better contrast, pictures were pseudocoloured in red (for CMA_3_) and green (for DAPI). Open arrows indicate CMA_3_-positive sites whose interindividual site-number variability is depicted in insets. Bar = 10 μm.

CMA_3_ labelled GC-rich regions associated exclusively with 45S rDNA sites in chromosomes of majority of species (Fig 4, S1B and S2 Figs). Exceptionally, CMA_3_- positive signal was found embedded in a single major locus of 5S rDNA site, along with other CMA_3_^+^ regions non-related to rDNA in selected specimen of *Ch*. *macracanthus* (S3A and S3D Fig). In genome of *S*. *superciliaris*, four out of six CMA_3_^+^ regions did not correspond either to 5S or 45S rDNA sites (S3H and S3J Fig). Slight intraspecific variability was recorded in number of CMA_3_^+^ sites in complements of *B*. *almorhae* (2 or 3), *L*. *guilinensis* (2, 3 or 4), *L*. *microphthalma* (4 or 5) and *S*. *pulchra* (4, 5 or 8) (Fig 4 and S3F Fig). In most cases, this feature mirrored the variability of 45S rDNA sites within respective genomes as revealed by FISH, while in one *S*. *pulchra* individual with eight CMA_3_^+^ sites (Fig 4D), this association remained inconclusive.

#### FISH with 5S, 28S rDNA and U2 snDNA probes

**Fig 5:**
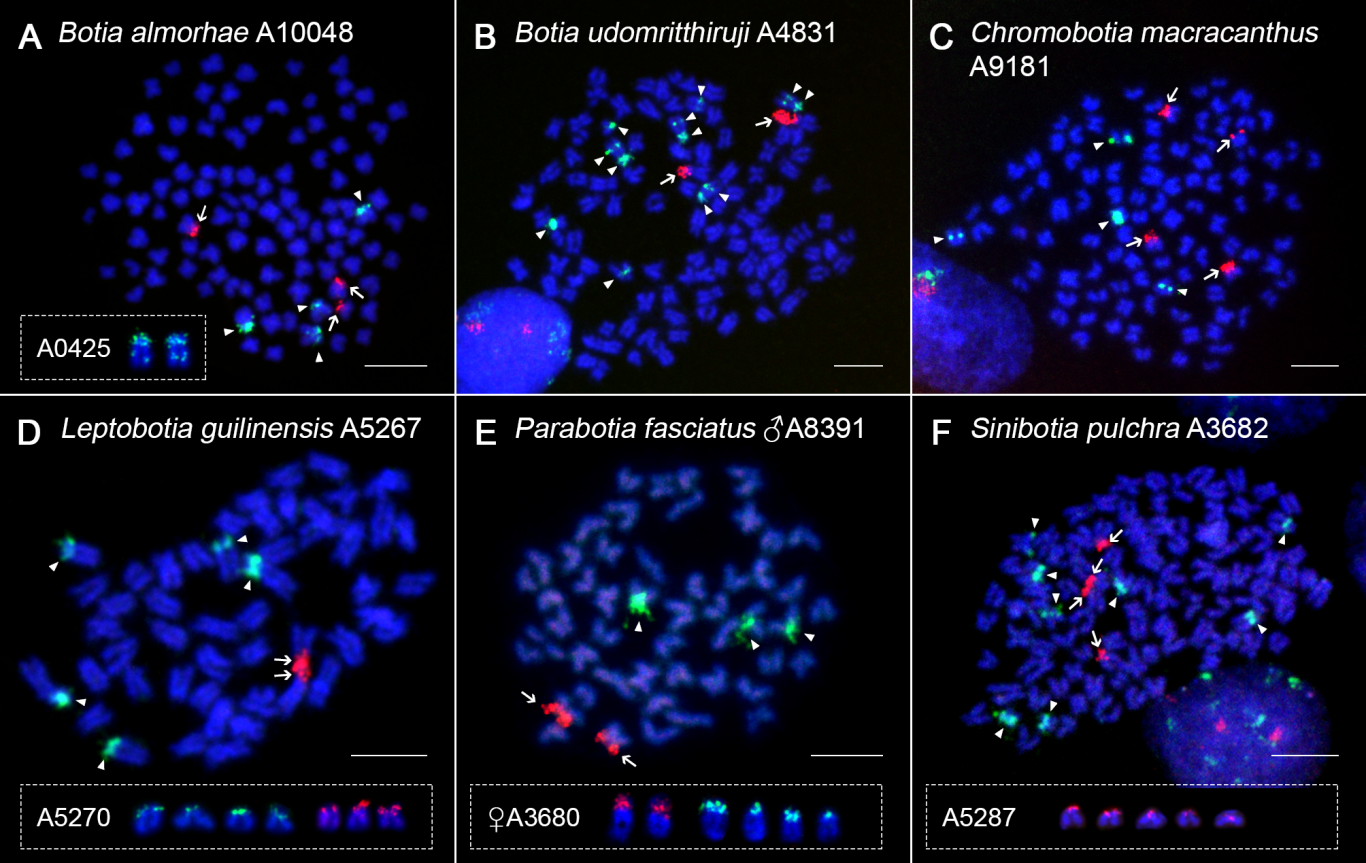
rDNA FISH in selected botiid species. 28S rDNA (red, arrows) and 5S rDNA (green, arrowheads) probes mapped on mitotic chromosomes of (**A**) *B*. *almorhae*, (**B**) *B*. *udomritthiruji*, (**C**) *Ch*. *macracanthus*, (**D**) *L*. *guilinensis*, (**E**) *P*. *fasciatus*, (**F**) *S*. *pulchra*. Chromosomes were counterstained with DAPI (blue). Note the presence of double sites of the 5S rDNA on one chromosomal pair in *B*. *almorhae* (**A**-inset) and two chromosomal pairs in *B*. *udomritthiruji* (**B**). Polymorphic rDNA sites in *L*. *guilinensis* (**D**), *P*. *fasciatus* (**E**) and *S*. *pulchra* (**F**) are boxed. Bar = 10 μm.

Partial idiograms showing rDNA phenotypes (i.e., numbers and position of rDNA clusters) and distribution of U2 snDNA sites in the phylogenetic context are summarized in Fig 2. The number of 45S rDNA clusters identified based on mapping of 28S rDNA probe was found in tetraploids to range from two to five. Remarkably, while several tetraploids (*B*. *dario*, *B*. *udomritthiruji*, *S*. *superciliaris*, *S*. *zebra*) showed only two 45S rDNA loci (Fig 5B, S1C, S4C and S4D Figs), the diploids displayed rather elevated site numbers of this rDNA class—up to four in *L*. *guilinensis* and even six in *L*. *elongata* (Figs 4B and 5D and S4A Fig); with up to five sites in *L*. *microphthalma* (deduced from CMA_3_ pattern; Fig 4C). The position of 45S rDNA cistrons along chromosomes was exclusively terminal in all specimens examined.

**Fig 6:**
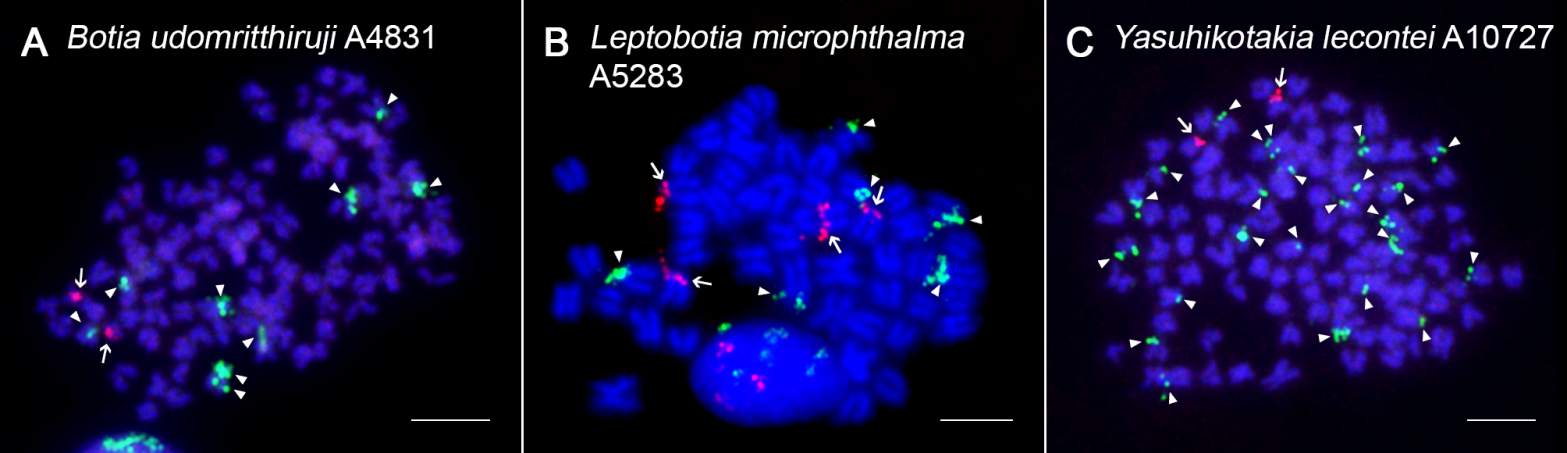
5S rDNA and U2 snDNA dual-colour FISH in selected botiid species. U2 snDNA (red, arrows) and 5S rDNA (green, arrowheads) probes mapped on mitotic chromosomes of (**A**) *B*. *udomritthiruji*, (**B**) *L*. *microphthalma*, (**C**) *Y*. *lecontei*. Chromosomes were counterstained with DAPI (blue). Note the significant spreading of 5S rDNA sites in *Y*. *lecontei* (**C**; 24 signals—arrowheads). The metaphase spread of *B*. *udomritthiruji* displays incomplete chromosome set (2n = 97), however the number of two U2 snDNA-bearing chromosomes was consistently observed on all other (including complete though less representative) metaphases in our dataset and with respect to Fig 5B, the number of 5S rDNA-bearing chromosomes is also complete. Bar = 10 μm.

5S rDNA clusters were mainly located in pericentromeric regions or distributed along the entire short (*p*) arms of some st-a chromosomes (Figs 5 and 6; S1C, S3A, S3H, S4, S5B, S5F and S5H Figs). Both in diploids and tetraploids we found generally higher number of 5S rDNA sites than encountered for the 45S rDNA class. The number of 5S rDNA FISH signals ranged from three to eight in majority of species (Figs 2 and 5 and S1C and S4 Figs), but also a sole intense signal was found in one specimen of *Ch*. *macracanthus* (S3A and S5B Figs). In *B*. *almorhae* and *B*. *udomritthiruji*, two and four st chromosomes, respectively, showed double 5S rDNA sites, with the major one terminally-located and the minor dot-like site detected in the interstitial position (Fig 5A and 5B). The minor sites were not always visible–mostly the chromosomes from the fin clipping procedure gave a greater resolution as they appeared more decondensed. Likewise, some metaphases allowed identifying intriguing organization of two tandem blocks of 45S rDNA regions on just one chromosome in *Y*. *lecontei* (S4E Fig).

We also found exceptional variability of multiplied 5S rDNA sites in a single species *Y*. *lecontei*, where one specimen displayed at least 13 5S rDNA signals after FISH (with the range of 13–16) (S4E Fig), while the second exhibited from 17 up to 24 clusters (Fig 6C). Multiplied 5S rDNA sites were located almost exclusively in terminal parts of st-a chromosomes. The intensity of signals was rather similar in both *Y*. *lecontei* specimens.

Further, slight intraspecific variability in the number of 5S and/or 45S rDNA was observed also in other botiid species under study–particularly in *Ch*. *macracanthus*, *P*. *fasciatus* (5S rDNA; Fig 5C and 5E and S3A and S5B Figs), *B*. *almorhae*, *L*. *microphthalma*, *S*. *pulchra* (45S rDNA; Figs 4A, 4C, 5A and 5F and S4B Fig) and in *L*. *guilinensis* (both rDNA classes, Figs 4B and 5D and S3F Fig). *P*. *fasciatus* was the only species in which the sex of the examined specimens was determined, and both sexes showed different rDNA phenotypes (Fig 5E). Additionally, size polymorphism between homologous 45S rDNA clusters as well as the presence of major vs. minor 5S rDNA loci displaying different size and signal strenght was apparent (e.g., Figs 4A, 4C and 5A–5C and S1C, S2D–S2F, S3C, S4A and S4E Figs). With respect to signal intensities, few metaphases in *Ch*. *macracanthus* specimen No. A9826 (i.e., the one with a single intense 5S rDNA site) suggested a putative presence of additional two-to-three minor dot-like sites, however, due to inconsistency of these observations we leave this issue inconclusive.

By contrast, U2 snDNA showed identical distribution on one pair of chromosomes both in diploids and tetraploids (Fig 6, S1D and S5 Figs), with a sole exception of diploid species *L*. *microphthalma* (four FISH signals; Fig 6B).

Based on combination of sequential experiments together with comparative analysis of morphology of rDNA/U2 snDNA-bearing chromosomes, none of the FISH probes derived from these distinct classes of multigene families displayed overlapping signals. Therefore, these motifs represent independent chromosomal markers due to their location on distinct chromosomes in tested species.

#### Telomeric FISH

**Fig 7:**
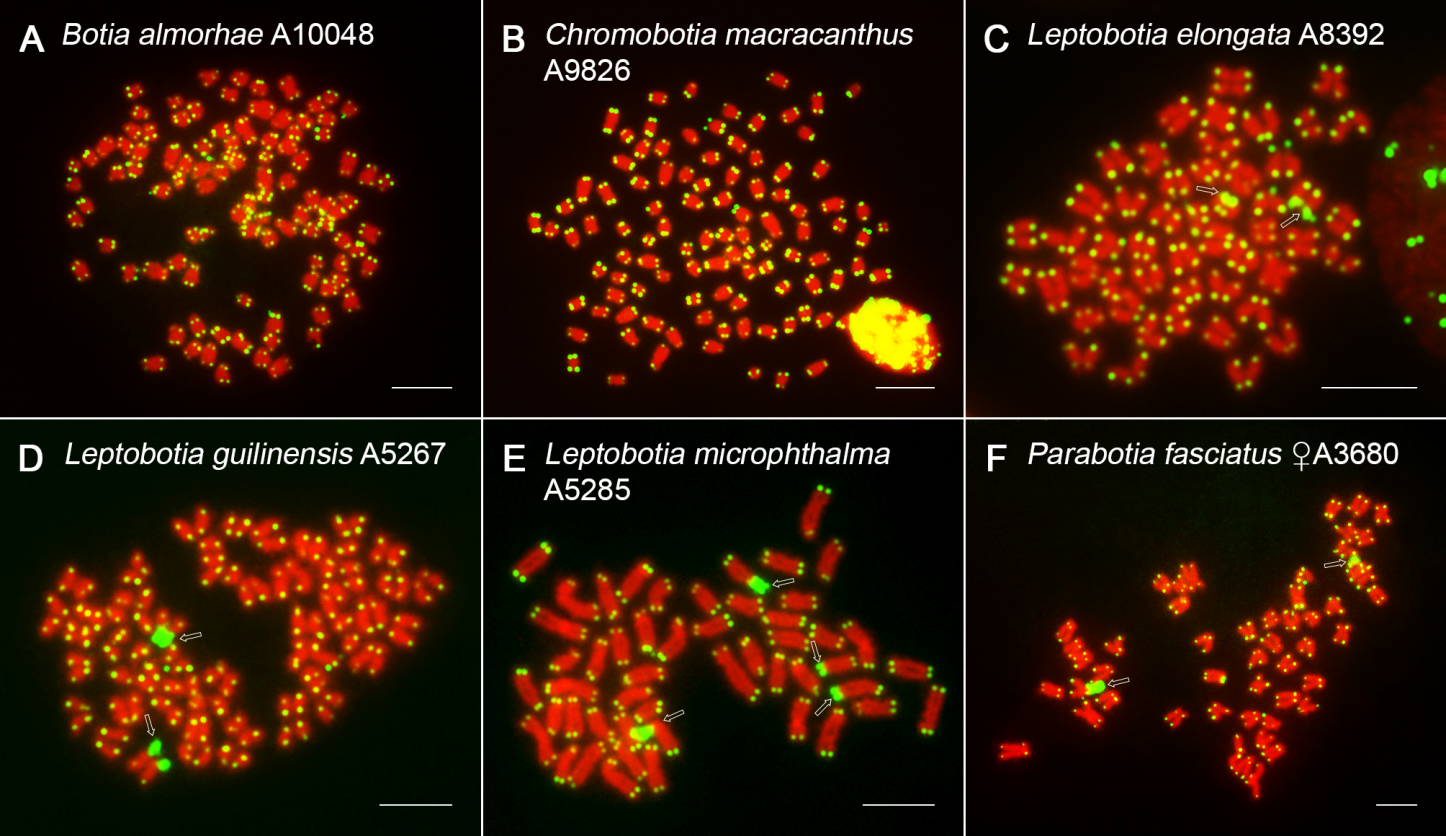
PNA FISH with telomeric probe in selected botiid species. Mitotic metaphases of (**A**) *B*. *almorhae*, (**B**) *B*. *udomritthiruji*, (**C**) *Ch*. *macracanthus*, (**D**) *L*. *guilinensis*, (**E**) *P*. *fasciatus*, (**F**) *S*. *pulchra*. For better contrast, pictures were pseudocoloured in green (telomeric repeat probe) and red (DAPI). Open arrows point to chromosomes with remarkable large-sized telomeric signals (**C-F**). Bar = 10 μm.

The FISH with a probe complementary to the conserved vertebrate (TTAGGG)_*n*_ telomeric motif revealed exclusively terminal location in tetraploid botiid species, with no detectable additional interstitial telomeric sequences (ITSs) (Fig 7, S1E and S6 Figs). Remarkably, no ITSs (and neither any other of specific repetitive DNA classes analyzed here) were present even in prominent DAPI^+^/AT-rich centromeric regions of large-sized m chromosomes in *B*. *dario* (S1E Fig).

In contrast, genomes of all analyzed diploid species shared the presence of several large-sized telomeric signals located terminally and interspersed with some or all 45S rDNA sites (Fig 7C–7F). Based on sequential experiments, two such ITS-like signals were found in *L*. *elongata* to be allocated within two non-homologous 45S rDNA sites, with the remaining four rDNA clusters lacking this association (S3B and S3E Fig). By contrast, a single pair of 45S rDNA signals found in one *L*. *guilinensis* individual entirely overlapped with the corresponding ITS-like sites (S3C and S3F Fig) and similar condition was observed in *L*. *microphthalma* specimen with four 45S rDNA loci (S3G and S3I Fig). Finally, based on the comparisons of non-sequential FISH and CMA_3_/DAPI staining results in *P*. *fasciatus*, this last diploid species under study does not seem to be exceptional regarding this observed peculiarity (Figs 5E and 7F,S2C Fig).

## Discussion

### Update on karyotype of botiid loaches

Out of 58 recognized species of botiid loaches, the number of those cytogenetically studied attained at present 34 ([44,47–49,73,74] and references therein) including new karyotype description of three species reported herein (*B*. *almorhae*, *B*. *udomritthiruji*, *L*. *microphthalma*) and three species (*B*. *histrionica*, *Syncrossus reversa*, *Yasuhikotakia nigrolineata*) where only 2n had been reported formerly [42]. The karyotypes of six species revised here (*B*. *dario*, *Ch*. *macracanthus*, *L*. *elongata*, *P*. *fasciatus*, *S*. *superciliaris* and *Y*. *lecontei*) differed to various extent from those in previous reports. While the karyotype of *L*. *elongata* in our analysis agreed well with a previous report of Yu et al. [75], those of *Ch*. *macracanthus*, *P*. *fasciatus* and *Y*. *lecontei* deviated slightly from former descriptions [48,74,75] due to different morphological classification of some chromosomal pairs resulting likely from difficulties associated with small size of botiid chromosomes and their gradual transitions both in size and centromere position. Finally, the karyotypes of *B*. *dario* (2n = 96) and *S*. *superciliaris* (2n = 100) described here are not consistent with those reported by Rishi and Haobam [76], Khuda-Bukhsh et al. [77] and Yue et al. [47] even in terms of 2n as the previous works described 2n = 90 or 98 for *B*. *dario* and 2n = 96 in *S*. *superciliaris*, respectively. The observed incongruences may have resulted from the description of karyotypically different populations or by mis-determination of some species in the earlier studies.

All but one tetraploid botiid loach species under study retained 2n that corresponds to their ploidy level, i.e., 2n = 4x = 100, implying no major interchromosomal rearrangements following the polyploidization event detectable by conventional staining. The only exception was *B*. *dario*, with derived karyotype comprising 2n = 96. Accordingly, all four diploid botiids under study shared the same 2n = 50 chromosomes and nearly identical NF. Karyotypes composed of 50 chromosomes are consistently found also in other loach families like Balitoridae [74], Cobitidae [44,78], Nemacheilidae ([53,79] and references therein), and Vaillantelidae [80], and they are also typical for many members of the suborder Cyprinoidei–a sister clade to loach groups [44,81]. Therefore, 2n = 50 may represent the putative ancestral state for Cypriniformes and its wide distribution across the whole group indicates its strong evolutionary conservatism.

Apart from the subtle karyotype changes, the generally reduced chromosomal size in tetraploid botiids compared to their diploid counterparts might reflect the commonly observed process of genome downsizing, with deletions through ectopic recombination acting as the main underlying mechanism [20,25,82]. In addition to these predicted deletions, also pericentric inversions along with other types of centromeric shifts (such as centromere repositioning; [83]) can alter chromosomal morphology without affecting 2n and thus might be responsible for interspecific differences in tetraploids' NF. Moreover, multiple inversions have been invoked as the postzygotic reproductive barrier contributing to speciation and adaptation [84,85], also in several fish groups (e.g., [86–89]) and they often largely contribute to gradual structural and sequence divergence of homologous or homeologous chromosomes within a re-diplodizing polyploid genome [90,91]. Finally, we also cannot rule out the possible involvement of reciprocal or non-reciprocal translocations, however the sound evidence for these rearrangements is missing in our study. In contrary, the indications for the origin of derived karyotype of *B*. *dario* through series of Robertsonian (Rb) translocations are strong, as the four largest elements of its karyotype are of metacentric morphology and they exhibit twice the size of average uni-armed element found within the complement. It is therefore highly likely that Rb translocations involving eight uni-armed chromosomes gave rise to the observed number of large-sized metacentrics.

### Physical mapping of chromosomal markers

#### Chromosomal distribution of CMA_3_^+^/GC-rich regions

CMA_3_-positive (CMA_3_^+^) sites confined to major 45S rDNA sites, i.e., nucleolar organizer regions (NORs) represent the prevailing pattern observed in species investigated here, matching the trend previously identified in overwhelming majority of fish species across the teleost phylogeny [92,93]. Similarly to some members of the related loach families Cobitidae [94–96] and Nemacheilidae [53], genomes of three species (*Ch*. *macracanthus*, *S*. *superciliaris* and *S*. *pulchra*) showed additional CMA_3_^+^ sites non-related to rDNA. This pattern was occasionally found also in other fish groups (e.g., [97–99]). Furthermore, just one conspicuous CMA_3_^+^ site from those detected in one specimen of *Ch*. *macracanthus* (subjected to sequential analysis of CMA_3_/DAPI staining and rDNA FISH) was embedded within a single major 5S rDNA site–an arrangement that is otherwise infrequent among fishes ([53,100] and references therein). Small intraspecific variation in the distribution of CMA_3_^+^ sites in *B*. *almorhae*, *L*. *guilinensis*, *L*. *microphthalma* and *S*. *pulchra* reflected variable NOR phenotypes and corroborated similar or higher intraspecific variability of this marker found in other loach fishes [53,95,96].

#### Differential patterns of variability in genomic organization and distribution of 5S, 45S rDNA and U2 snDNA

The mapping of tandemly repeated sequences by means of FISH has proven to be very useful in exploring fish genome architecture as well as large amount of evolutionary, ecological and taxonomic questions [101–103]. In this study, three tandemly repeated multigene families– 5S/45S rDNA and U2 snDNA–were used to compare diploid vs. polyploid genome dynamics in botiid loaches.

In most fishes [104–106], including other loach families [51–53,55] the ancestral diploid pattern appears to be one pair of NOR/45S rDNA sites and the emerging information suggest similar scenario for U2 snDNA. In 5S rDNA, however, the situation has been found to be too variable and complex to reconstruct the ancestral situation ([106,107]; for examples in loaches and cypriniform polyploids, see: [30,33,50,53,55,108]). Contrary to expectations, our present results show that the number of 45S rDNA signals range from two to six in diploid botiids, thereby exceeding frequently the expected number. In contrast, the 45S rDNA phenotypes in tetraploids exceeded the corresponding expected number of four clusters only in one specimen of *S*. *pulchra* (five signals). Moreover, four out of eight tetraploid botiid species displayed only two 45S rDNA signals instead of the expected four. This site-number reduction might reflect the gradual processes leading to elimination of excessive rDNA clusters in (especially ancient) polyploids.

FISH with minor (5S rDNA) ribosomal cluster unveiled generally higher and more variable number of sites compared to 45S rDNA, similarly to those found in nemacheilid loaches [53] and other cypriniforms (e.g., [30,33]), while yet another related groups display the inverse situation (i.e., high number of variable 45S rDNA sites vs. conservative pattern with low site-number of 5S rDNA [27,50]).

An unusual pattern was observed in *B*. *almorhae* and *B*. *udomritthiruji*, where 5S rDNA site turned out to be partially shifted from a terminal to interstitial position (or *vice versa*) in one (*B*. *almorhae*) and two (*B*. *udomritthiruji*) pairs of st chromosomes, creating thus double 5S rDNA site. Similarly to what has been proposed in other (not only) fish groups ([109] and references therein), a peri- or paracentric inversion might have mediated the transfer of few 5S rDNA transcriptional units from the original cluster to a new location. However, especially considering the large size of such 5S rDNA-bearing chromosomes, even reciprocal or non-reciprocal translocations cannot be currently excluded from consideration.

Associations of rDNAs with para/pericentric inversions in fishes have been repeatedly documented [53,88,97,109–111] and might also be responsible for the emergence of large-sized tandemly organized 45S rDNA cluster on just one homolog in *Y*. *lecontei* (as in the scenario proposed in Ghigliotti et al. [112]), being clearly coupled with tandem amplifications driven by unequal crossing-overs [112–114]. The latter mechanism is, together with possible reciprocal translocations between the rDNA loci and subsequent random segregation of rDNA-bearing chromosomes in meiosis, highly likely responsible for size heteromorphism of both rDNA clusters and for subtle site-number polymorphisms repeatedly observed in other botiids under study regardless their ploidy level (as well as in many other fish groups). In some cases, if not entirely deleted, the amount of rDNA sequences might be reduced below the resolution level of FISH, which might explain, for instance, the 5S rDNA intraspecific site-number variability in *Ch*. *macracanthus*. In *P*. *fasciatus*, despite the site-number polymorphism of 5S rDNA is associated with the sex of the analyzed specimens, the undersampling does not permit us to infer any meaningful conclusions about the presence of cryptic sex chromosomes.

One of the most intriguing results of our study is the tremendously increased and variable number of 5S rDNA sites in the tetraploid species *Y*. *lecontei*. Over the last decade, many studies have provided evidence of rapidly amplified loci for both major and minor rDNA class in fishes ([106,107] and references therein) including both nascent and ancient polyploids [28,31,33,98]. Several mechanisms have been put forward to explain this phenomenon including i) unequal crossing-over and ectopic recombination ii) activity of transposable elements (TEs), iii) extrachromosomal replication and reintegration via extrachromosomal circles of rDNA or combined effect of the three (for comprehensive discussion, see: [115,116]). While the terminal distribution of 5S rDNA sites in *Y*. *lecontei* might favour the first possibility [116,117], the significant difference in the loci number of 5S rDNA between the two analyzed individuals might argue in favour of rDNA dispersion through the action of TEs, as being repeatedly indicated in teleosts through FISH and/or DNA sequence analysis ([53,57,106,115,118–120] and references therein).

Small nuclear RNAs (snRNAs; U1, U2, U4/U6 and U5) are indispensable components of spliceosome–a ribonucleoprotein complex responsible for removing introns from the vast majority of eukaryotic primary mRNA transcripts (reviewed in [121,122]). In recent years, U2 snDNA became a novel marker in fish cytogenetic studies. Together with species in our study, this tandemly repeated multigene family has been mapped already in about 80 species placed in ten fish orders (reviewed in [105]; among more recent papers, see, e.g.: [89,123–125]), with our study adding the first records for Cypriniformes. About half of the surveyed teleost fish species exhibits a single pair of U2-bearing chromosomes and internal or proximal chromosomal localization [89,105,124].

Contrary to expectations, FISH labelled only one chromosome pair bearing U2 snDNA in all but one botiid species regardless their ploidy level. As the only exception, the diploid species *L*. *microphthalma* displayed four signals. Such result confirms, on one hand, the high conservatism of chromosomal distribution of this multigene family and, on the other hand, it also indicates advanced stage of genome homogenization and re-diploidization in tetraploid botiids.

To sum up, our results unmasked differential molecular drives in three multigene families, with increasing variability in the direction U2 snDNA < 45S rDNA < 5S rDNA, implying various extent and degree of interplay between two broadly accepted models of long-term repetitive DNA molecular evolution (i.e., concerted and birth-and-death evolution [29,32,107,126–130]). The observed patterns do not reflect the phylogenetic relationships of the species, neither their ploidy level; which hampers the exact identification of the driving forces at present. However, the hybridization patterns of multigene families collectively imply a significant re-diploidization in genomes of tetraploid botiids and thus corroborate the view of an old and rather single WGD event at the base of Leptobotiinae/Botiinae split. This is particularly noticeable on fully re-diploidized U2 snDNA sites, while observed gains and losses in rDNA loci indicate more complex evolutionary dynamics. Cases of intraspecific polymorphisms and rDNA amplification together with evidence of associated chromosome rearrangements may suggest a role of repetitive DNA in dynamic processes leading to post-polyploid genome divergence and possibly to speciation. The current dataset along with the supposed age of botiid WGD makes it, on the other hand, difficult to draw any conclusions about the origin of this polyploidization (i.e., whether it was autopolyploidy or allopolyploidy). Despite some previous studies [48] presumed an autopolyploidization as the underlying mechanism using conventionally stained chromosomes only, it remained unclear on which facts they built this conclusion. Current data are, however, sufficient enough to reinforce the conclusion from our previous study [49] that *S*. *zebra* is not a hybrid between the tetraploid species *S*. *pulchra* and the diploid species *L*. *guilinensis*.

#### Inferences from distribution of telomeric (TTAGGG)_*n*_ sequences

In all tetraploid botiid species, FISH with probe complementary to telomeric (TTAGGG)_*n*_ repeats showed signals only in their usual location at termini of all chromosomes. Interstitial telomeric sequences (ITSs), representing often hallmarks of chromosomal repatternings during the course of karyotype evolution (reviewed in [131,132]), were not observed. Neither in *B*. *dario* with karyotype derived by Rb translocations, where only large tandemly arranged AT-rich/DAPI-positive blocks were apparent in the fusion points. Remarkably, all analyzed diploid species displayed large terminal blocks of telomere-like sequences, all of them being interspersed with NORs/45S rDNA sites. A similar pattern was previously described in genomes of several fish groups ([53,132–134] and references therein) as well as in other evolutionarily distant organisms (e.g., [131,135–137]). The evolutionary significance of this merged and co-amplified rDNA/telomeric arrangement is still under debate, however, the accumulating evidence of this phenomenon deserves greater attention ([135–137] and references therein). It has been suggested that major rDNA sites might play a supportive role in stabilization of chromosomal termini and, reciprocally, telomeres might be involved in nucleolar organization ([135,138]; for more references, see [134]). Besides that, telomeric position effect on NOR expression had been considered (e.g., [139]), but no disruption of NOR activity was documented in some other studies [53,135]. Neither our results are consistent with this hypothesis, since some diploid botiids (at the species or individual level) showed all 45S rDNA regions being entirely co-localized with (TTAGGG)_*n*_ repeats, with at least some of them evidently retaining the transcriptional activity. By analogy, the presence of various repetitive DNA classes embedded within rDNA clusters does not seem to negatively interfere with the transcription activity of NORs in some other fishes ([37,140,141] but see [142,143]). Finally, the stretches of co-amplified telomeric/rDNA repeats might have occurred rather as a consequence of TE activity and double-strand break repair mechanisms utilizing several recombinational pathways and *de novo* DNA synthesis [144,145], especially when considering the high recombinogenic potential of both involved tandemly repeated DNA classes [117,145].

### Phylogenetic interpretation of the molecular cytogenetic markers

When the number and position of 5S/45S rDNA and U2 snDNA sites were mapped onto our phylogenetic tree, almost no correlation between the cytogenetic markers and the phylogenetic relationships of the species was apparent (Fig 2), implying mostly different evolutionary trajectories, unrelated and independent with respect to evolution of this fish group and corroborating findings from some other teleosts [106,118]. Surprisingly, diploid botiids appear to have gone through more pronounced changes of rDNA phenotypes in terms of polymorphisms and gaining new rDNA sites than their tetraploid counterparts, except for *Y*. *lecontei* that exhibits a significant increase of amplified 5S rDNA loci. Interestingly, all diploid species shared this high repetitive DNA dynamics despite conservative karyotype macrostructure–similarly to nemacheilids [53] and several other fish groups (e.g., [87,146] and references therein).

In addition, all analyzed diploid species further shared remarkable large terminal blocks of telomere-like sequences, all of them being coincident with NORs/45S rDNA sites. Since only genomes of diploid species possessed such pattern, this arrangement likely arose after the divergence of Leptobotiinae from Botiinae, but before the split between *Leptobotia* and *Parabotia*. The alternative hypothesis that such arrangement was already present in ancestral botiid lineage before WGD, is less parsimonious, because even if rapid preferential elimination of conjugated rDNA/telomeric sites took place in polyploids, the probability of at least few of them being still preserved in some species is not negligible.

Within tetraploids, the sister species *B*. *almorhae* and *B*. *udomritthiruji* shared the presence of double sites of the 5S rDNA cluster on some chromosomes and a pericentromeric position of U2 snDNA in a small metacentric pair in contrast to the rest of the species under study, where the position of U2 snDNAs was found to be terminal, occupying *p*-arms of a given st-a chromosome pair. With regards to the occurrence of Rb translocations in the later *Botia* species included in the dataset, our analysis evidenced chromosomal rearrangements mainly in this tetraploid genus. In addition, 45S rDNA sites were probably homeologous between analyzed botiids, in contrast to variable 5S rDNA phenotypes.

## Conclusion

To sum up, the findings from the current study allowed us to reach following conclusions: i) selected cytogenetic markers showed different molecular drives, being in agreement with proposed modes of long-term molecular evolution of tandemly arrayed sequences, ii) the evolution of these markers does not seem to follow the phylogenetic relationships of studied botiids, ii) diploids showed unexpectedly higher dynamics of rDNA phenotypes compared to tetraploids, iii) individual tetraploid genomes followed distinct patterns of genome repatterning, but generally exhibit iv) high degree of rediploidization, with mosaic of diploid and tetraploid genomic regions in all studied polyploids, corroborating the view of already old-aged and rather single WGD event that predated Leptobotiinae/Botiinae divergence, especially with regards to uniformly re-diploidized number of U2 snDNA sites. Our findings thus point to intense post-polyploid genome dynamics and possible impact of repetitive DNAs on genome divergence. Finally, the current cytogenetic dataset will contribute to the follow-up research aimed at integrated in-depth cyto-genomic analysis addressing the mechanistic nature of botiid polyploidization event.

## Supporting information

S1 Fig Karyotype and mitotic chromosomes of *B*. *dario* after different cytogenetic protocols. (**A**) Karyotype arranged from Giemsa-stained chromosomes, (**B**) CMA_3_/DAPI staining. (**C**) Dual-colour FISH with 28S rDNA (red, arrows) and 5S rDNA (green, arrowheads) probes. (**D**) Uni-colour FISH with U2 snDNA (red, arrows) probe. (**E**) PNA FISH with telomeric probe. Due to low number of complete and/or well-spread metaphases, besides complete plates with 2n = 96 (**A**,**D**) also incomplete plates with 2n = 95 (**B**,**C**) and 2n = 91 (**E**) had to be selected, providing however sufficient data to present required features (e.g., absence of ITSs in large-sized metacentric chromosomes; **E**). For better contrast, images were pseudocoloured in red (for CMA_3_) and green (for DAPI) for CMA_3_/DAPI staining (**B**) and in green (telomeric repeat probe) and red (DAPI) for telomeric PNA FISH (**E**). Bar = 10 μm.
(TIF)

S2 Fig CMA_3_/DAPI staining in the rest of examined botiid species. (**A**) *B*. *udomritthiruji*, (**B**) *L*. *elongata*, (**C**) *P*. *fasciatus*, (**D**) *S*. *superciliaris*, (**E**) *S*. *zebra*, (**F**) *Y*. *lecontei*. For better contrast, pictures were pseudocoloured in red (for CMA_3_) and green (for DAPI). Open arrows indicate CMA_3_-positive sites. The metaphase spread of *S*. *superciliaris* (**D**) is incomplete (2n = 97), but the number of CMA_3_^+^ signals is congruent with S6F Fig. Bar = 10 μm.
(TIF)

S3 Fig Selected sequential experiments clarifying conjugated or independent location of distinct cytogenetic markers. Metaphases are arranged sequentially in *Ch*. *macracanthus* (**A**,**D**), *L*. *elongata* (**B**,**E**), *L*. *guilinensis* (**C**,**F**), *L*. *microphthalma* (**G**,**I**) and *S*. *superciliaris* (**H**,**J**) after CMA_3_/DAPI staining (**D,E,F,I,J**) and corresponding dual-colour FISH with 28S rDNA (red, arrows) and 5S rDNA (green, arrowheads) probes (**A,H**) or PNA FISH with telomeric probe (**B**,**C**,**G**). For better contrast, images were pseudocoloured in red (for CMA_3_) and green (for DAPI) for CMA_3_/DAPI staining and in green (telomeric repeat) and red (DAPI) for telomeric PNA FISH. Note the co-localization of single interstitial 5S rDNA site (**A**; open arrowhead) with prominent CMA_3_^+^ band (**D**) in Ch. *macracanthus*. Notice also partial overlap of extended telomere-like sequences (**B**; open arrows) with a set of CMA_3_^+^/45S rDNA sites in *L*. *elongata* (**E**; open arrowheads) and complete interspersion of these sequences in *L*. *guilinensis* (**C**,**F**) and *L*. *microophthalma* (**G**,**I**). Finally, in *S*. *superciliaris*, Figs **H** and **J** show correspondence between 45S rDNA (**H**; arrows) and CMA_3_^+^ sites (**J**; empty arrowheads), while completely independent locations of 5S rDNA (H; arrowheads) and CMA_3_^+^ sites (**J**; empty arrowheads) is clearly apparent. The site-numbers of particular markers on incomplete figures (**C**,**F**—*L*. *guilinensis*, 2n = 49; **G**,**I**–*L*. *microphthalma*, 2n = 49) can be verified on Fig 7D and 7E, respectively. Bar = 10 μm.
(TIF)

S4 Fig Dual-colour (5S/45S) rDNA FISH in the rest of examined botiid species. 28S rDNA (red, arrows) and 5S rDNA (green, arrowheads) probes mapped on (**A**) *L*. *elongata*, (**B**) *L*. *microphthalma*, (**C**) *S*. *superciliaris*, (**D**) *S*. *zebra*, (**E**) *Y*. *lecontei*. Chromosomes were counterstained with DAPI (blue). Note the significant spreading of 5S rDNA sites in *Y*. *lecontei* (**E**; 13 signals—arrowheads). On the same picture (*Y*. *lecontei*; **E**), two adjacent arrows point to tandemly arranged double-sided 45S rDNA site, scarcely detectable based on degree of chromosome condensation (compare with S2F Fig). Despite being incomplete (2n = 99), metaphase spread of *Y*. *lecontei* (**E**) displays complete number of 45S rDNA signals observed in this species (see S2F Fig) and the number of 5S rDNA signals fits the range observed in our dataset for this specimen. Bar = 10 μm.
(TIF)

S5 Fig FISH with 5S rDNA and U2 snDNA probes in the rest of examined botiid species. U2 snDNA (red, arrows) and 5S rDNA (green, arrowheads) probes (**B**,**F**,**H**) or a single U2 snDNA (red, arrows) probe (**A**,**C**,**D**,**E**,**G**) mapped on (**A**) *B*. *almorhae*, (**B**) *Ch*. *macracanthus*, (**C**) *L*. *elongata*, (**D**) *L*. *guilinensis*, (**E**) *P*. *fasciatus*, (**F**) *S*. *pulchra*, (**G**) *S*. *superciliaris*, (**H**) *S*. *zebra*. The metaphase spread of *S*. *zebra* (**H**) is incomplete (2n = 99). Chromosomes were counterstained with DAPI (blue). Bar = 10 μm.
(TIF)

S6 Fig PNA FISH with telomeric probe in the rest of examined botiid species. (**A**) *B*. *udomritthiruji*, (**B**) *S*. *pulchra*, (**C**) *S*. *superciliaris*, (**D**) *S*. *zebra*, (**E**) *Y*. *lecontei*. For better contrast, pictures were pseudocoloured in green (telomeric repeat probe) and red (DAPI). For better distinction between individual chromosomes, we included also the separate image with DAPI channel for *S*. *superciliaris* (**C**). Bar = 10 μm.
(TIF)
