# Supplementary figures and images for: Dynamics of tandemly repeated DNA sequences during evolution of diploid and tetraploid botiid loaches (Teleostei: Cobitoidea: Botiidae)
Source: PLoS One. 2018 Mar 28;13(3):e0195054. doi: 10.1371/journal.pone.0195054 (PMC5874072; doi:10.1371/journal.pone.0195054)

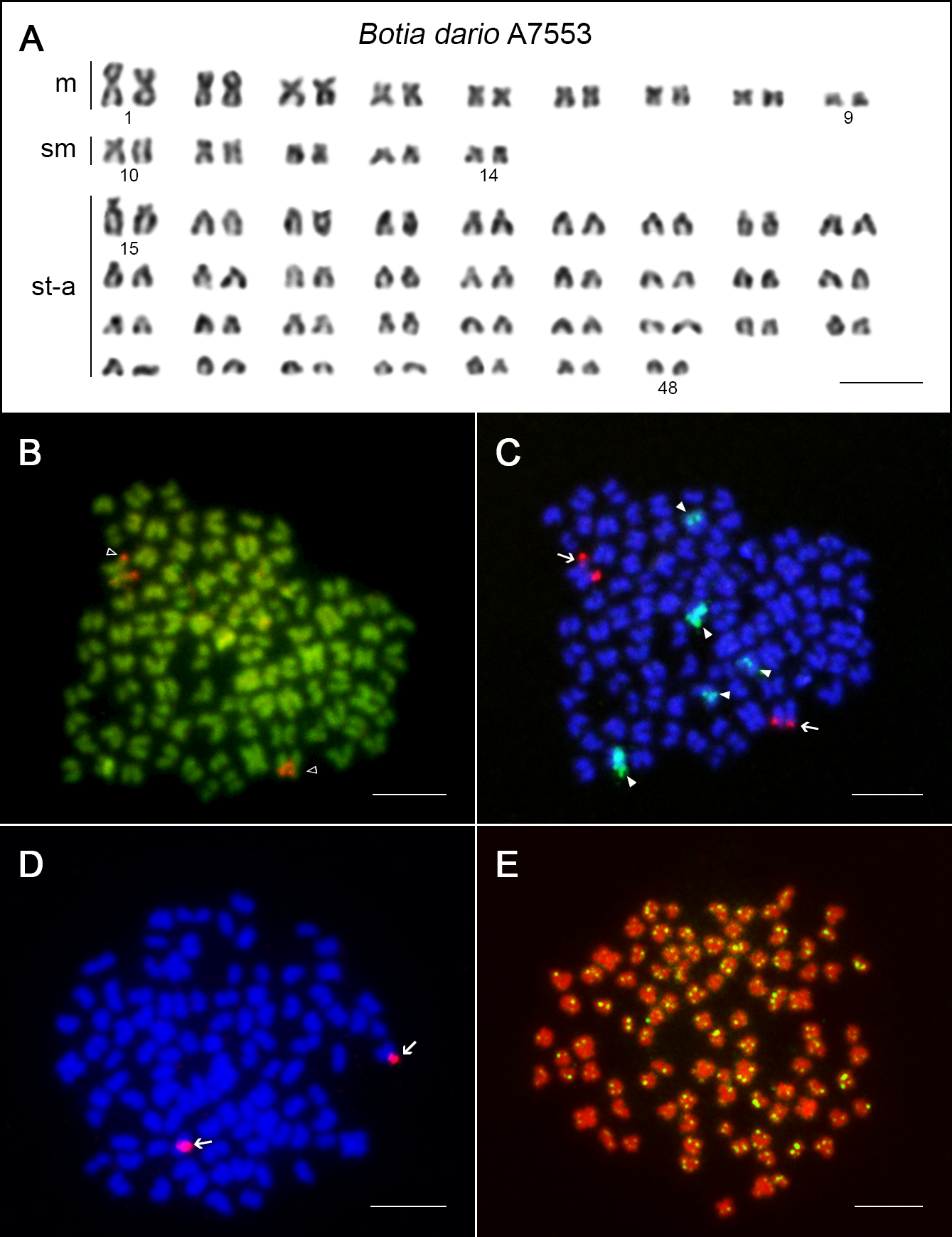

Supplement: S1 Fig — (A) Karyotype arranged from Giemsa-stained chromosomes, (B) CMA3/DAPI staining. (C) Dual-colour FISH with 28S rDNA (red, arrows) and 5S rDNA (green, arrowheads) probes. (D) Uni-colour FISH with U2 snDNA (red, arrows) probe. (E) PNA FISH with telomeric probe. Due to low number of complete and/or well-spread metaphases, besides complete plates with 2n = 96 (A,D) also incomplete plates with 2n = 95 (B,C) and 2n = 91 (E) had to be selected, providing however sufficient data to present required features (e.g., absence of ITSs in large-sized metacentric chromosomes; E). For better contrast, images were pseudocoloured in red (for CMA3) and green (for DAPI) for CMA3/DAPI staining (B) and in green (telomeric repeat probe) and red (DAPI) for telomeric PNA FISH (E). Bar = 10 μm. (TIF) [file pone.0195054.s001.tif]

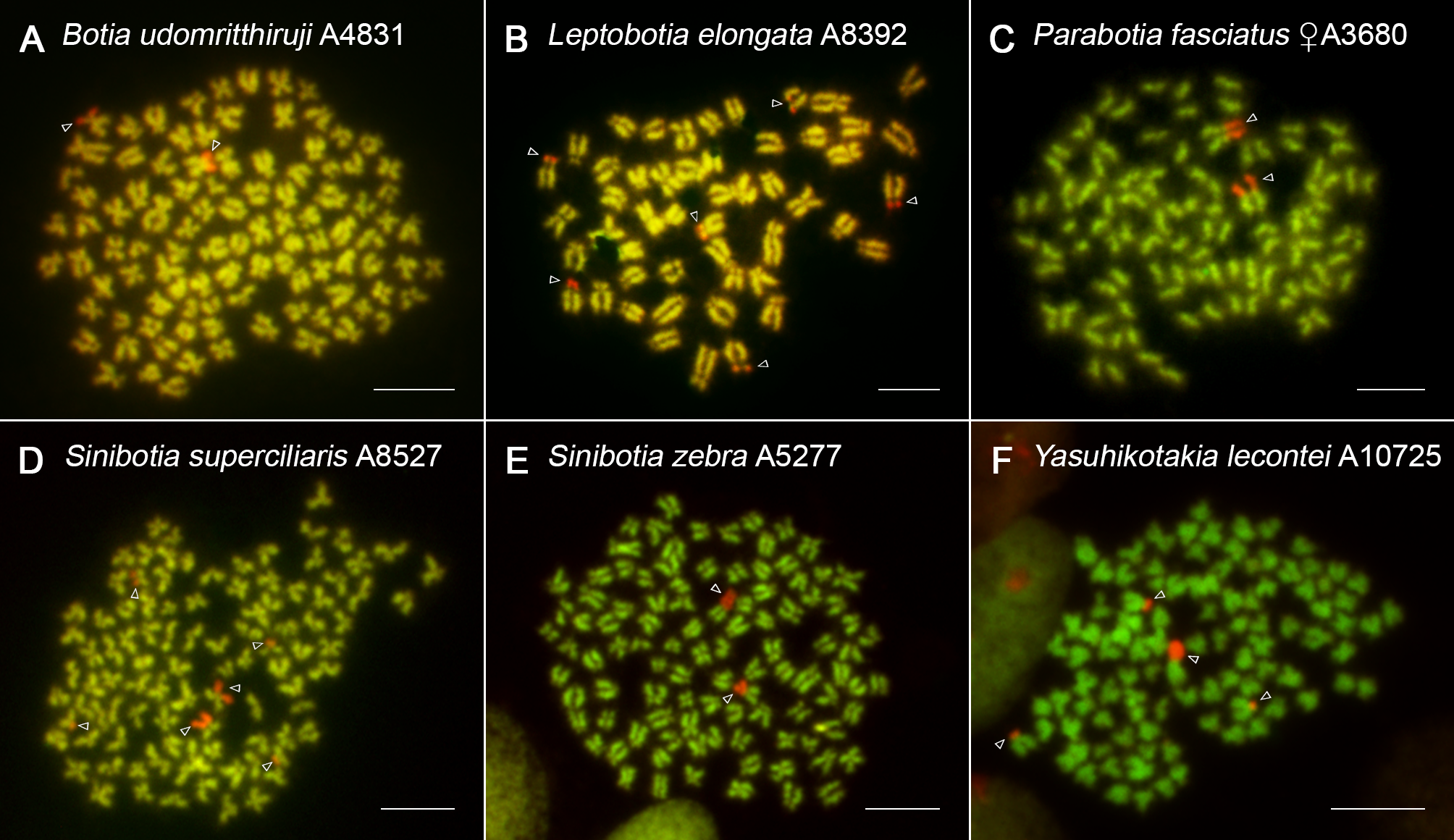

Supplement: S2 Fig — (A) B. udomritthiruji, (B) L. elongata, (C) P. fasciatus, (D) S. superciliaris, (E) S. zebra, (F) Y. lecontei. For better contrast, pictures were pseudocoloured in red (for CMA3) and green (for DAPI). Open arrows indicate CMA3-positive sites. The metaphase spread of S. superciliaris (D) is incomplete (2n = 97), but the number of CMA3+ signals is congruent with S6F Fig. Bar = 10 μm. (TIF) [file pone.0195054.s002.tif]

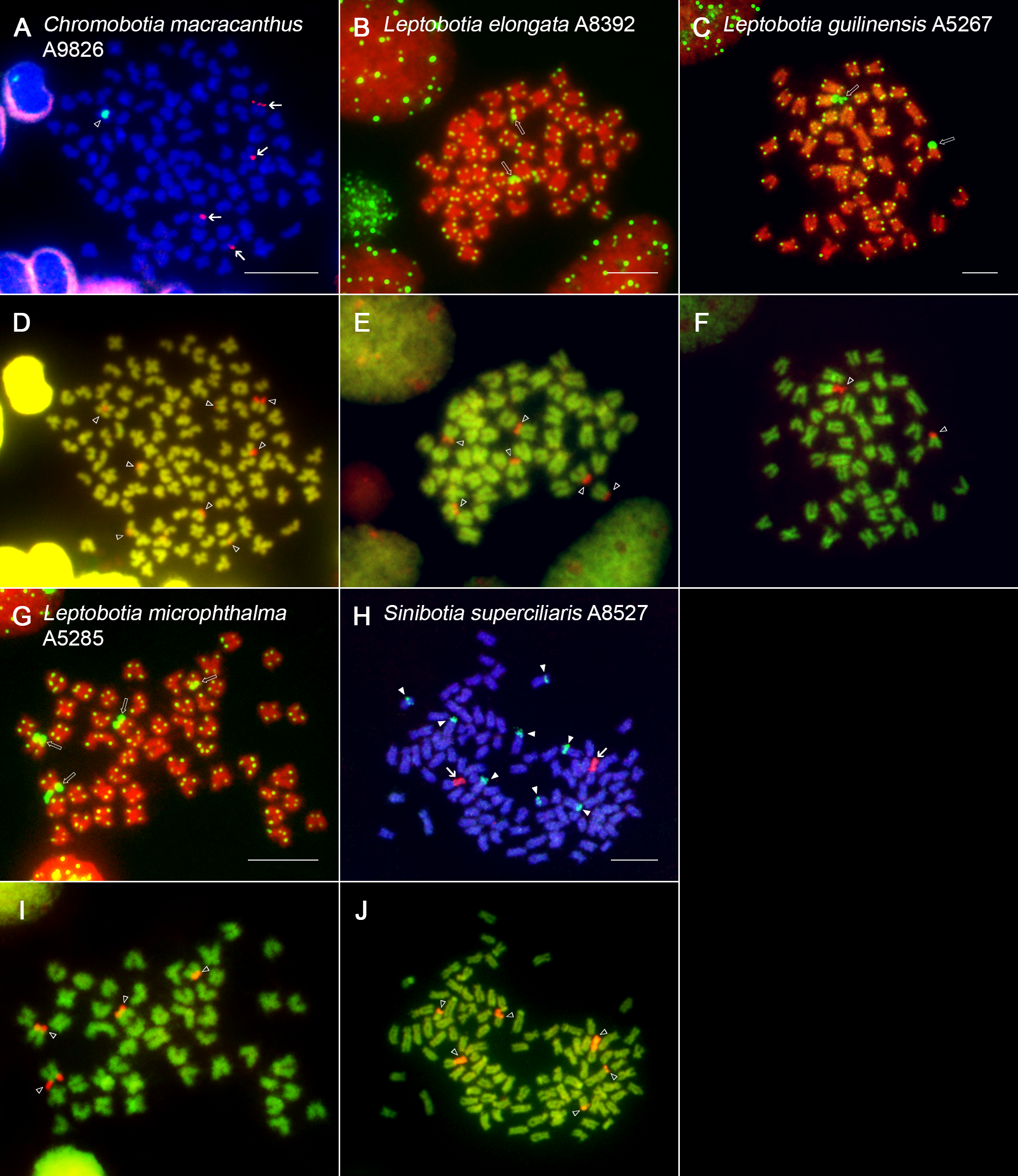

Supplement: S3 Fig — Metaphases are arranged sequentially in Ch. macracanthus (A,D), L. elongata (B,E), L. guilinensis (C,F), L. microphthalma (G,I) and S. superciliaris (H,J) after CMA3/DAPI staining (D,E,F,I,J) and corresponding dual-colour FISH with 28S rDNA (red, arrows) and 5S rDNA (green, arrowheads) probes (A,H) or PNA FISH with telomeric probe (B,C,G). For better contrast, images were pseudocoloured in red (for CMA3) and green (for DAPI) for CMA3/DAPI staining and in green (telomeric repeat) and red (DAPI) for telomeric PNA FISH. Note the co-localization of single interstitial 5S rDNA site (A; open arrowhead) with prominent CMA3+ band (D) in Ch. macracanthus. Notice also partial overlap of extended telomere-like sequences (B; open arrows) with a set of CMA3+/45S rDNA sites in L. elongata (E; open arrowheads) and complete interspersion of these sequences in L. guilinensis (C,F) and L. microophthalma (G,I). Finally, in S. superciliaris, Figs H and J show correspondence between 45S rDNA (H; arrows) and CMA3+ sites (J; empty arrowheads), while completely independent locations of 5S rDNA (H; arrowheads) and CMA3+ sites (J; empty arrowheads) is clearly apparent. The site-numbers of particular markers on incomplete figures (C,F—L. guilinensis, 2n = 49; G,I–L. microphthalma, 2n = 49) can be verified on Fig 7D and 7E, respectively. Bar = 10 μm. (TIF) [file pone.0195054.s003.tif]

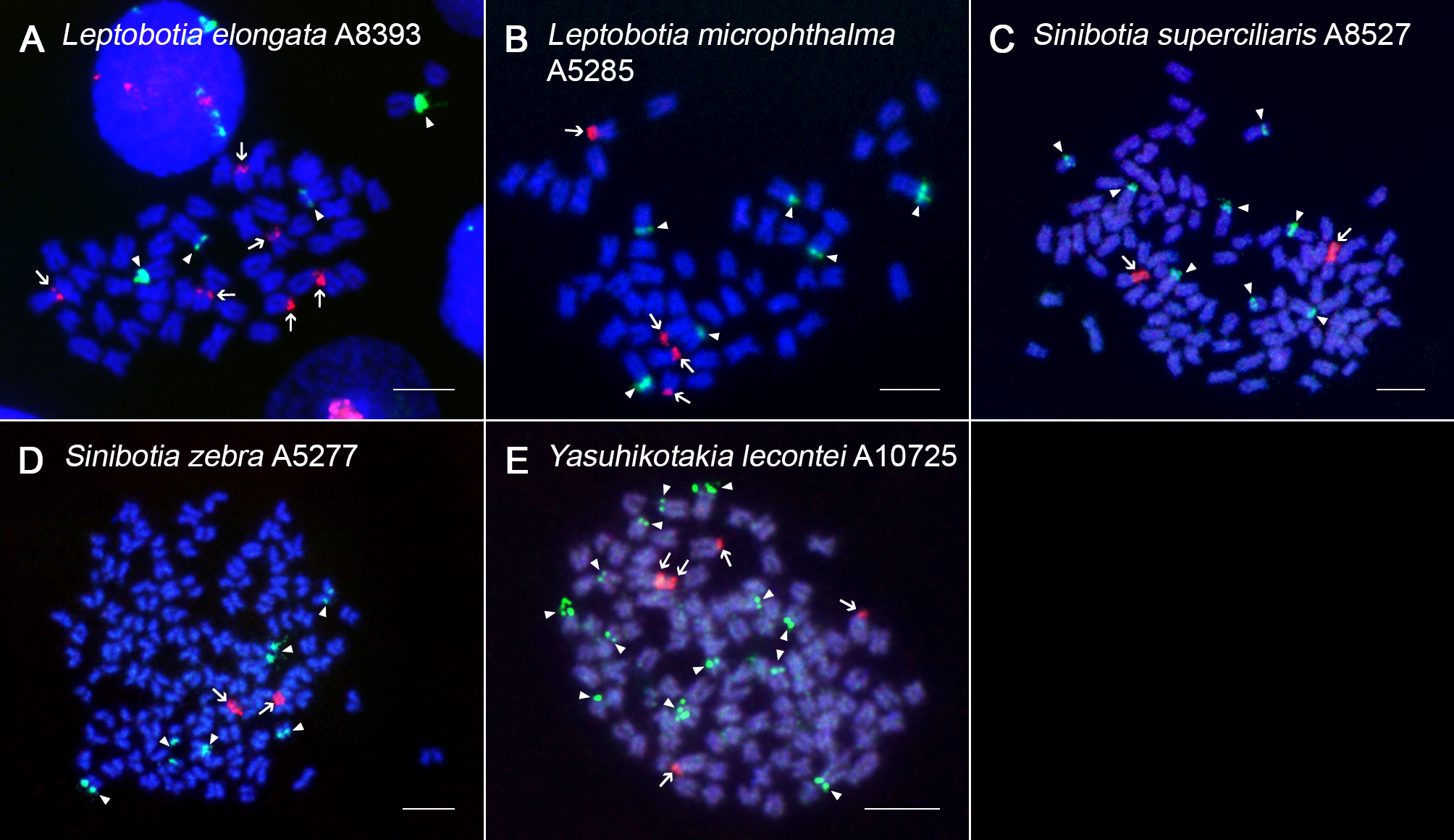

Supplement: S4 Fig — 28S rDNA (red, arrows) and 5S rDNA (green, arrowheads) probes mapped on (A) L. elongata, (B) L. microphthalma, (C) S. superciliaris, (D) S. zebra, (E) Y. lecontei. Chromosomes were counterstained with DAPI (blue). Note the significant spreading of 5S rDNA sites in Y. lecontei (E; 13 signals—arrowheads). On the same picture (Y. lecontei; E), two adjacent arrows point to tandemly arranged double-sided 45S rDNA site, scarcely detectable based on degree of chromosome condensation (compare with S2F Fig). Despite being incomplete (2n = 99), metaphase spread of Y. lecontei (E) displays complete number of 45S rDNA signals observed in this species (see S2F Fig) and the number of 5S rDNA signals fits the range observed in our dataset for this specimen. Bar = 10 μm. (TIF) [file pone.0195054.s004.tif]

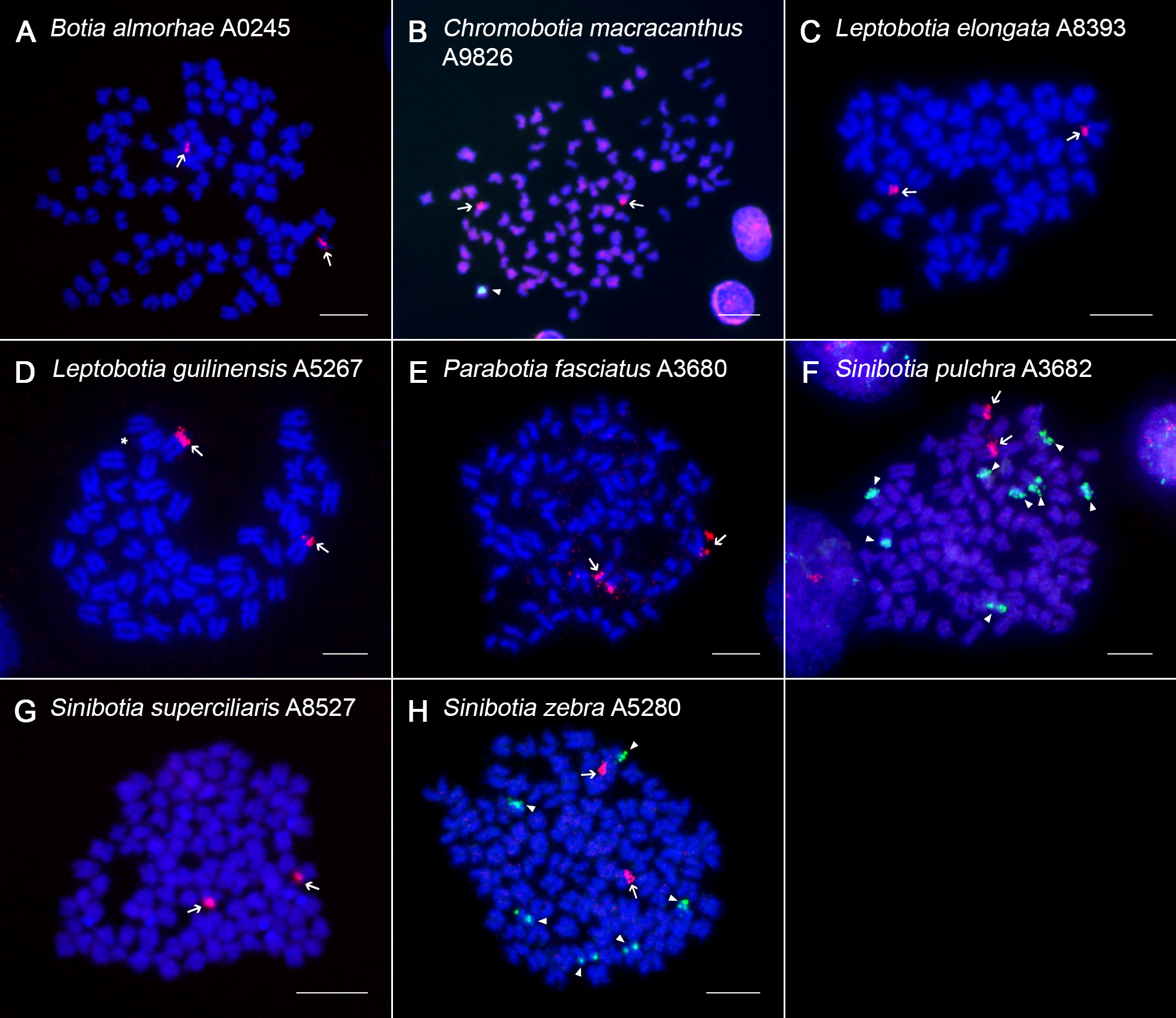

Supplement: S5 Fig — U2 snDNA (red, arrows) and 5S rDNA (green, arrowheads) probes (B,F,H) or a single U2 snDNA (red, arrows) probe (A,C,D,E,G) mapped on (A) B. almorhae, (B) Ch. macracanthus, (C) L. elongata, (D) L. guilinensis, (E) P. fasciatus, (F) S. pulchra, (G) S. superciliaris, (H) S. zebra. The metaphase spread of S. zebra (H) is incomplete (2n = 99). Chromosomes were counterstained with DAPI (blue). Bar = 10 μm. (TIF) [file pone.0195054.s005.tif]

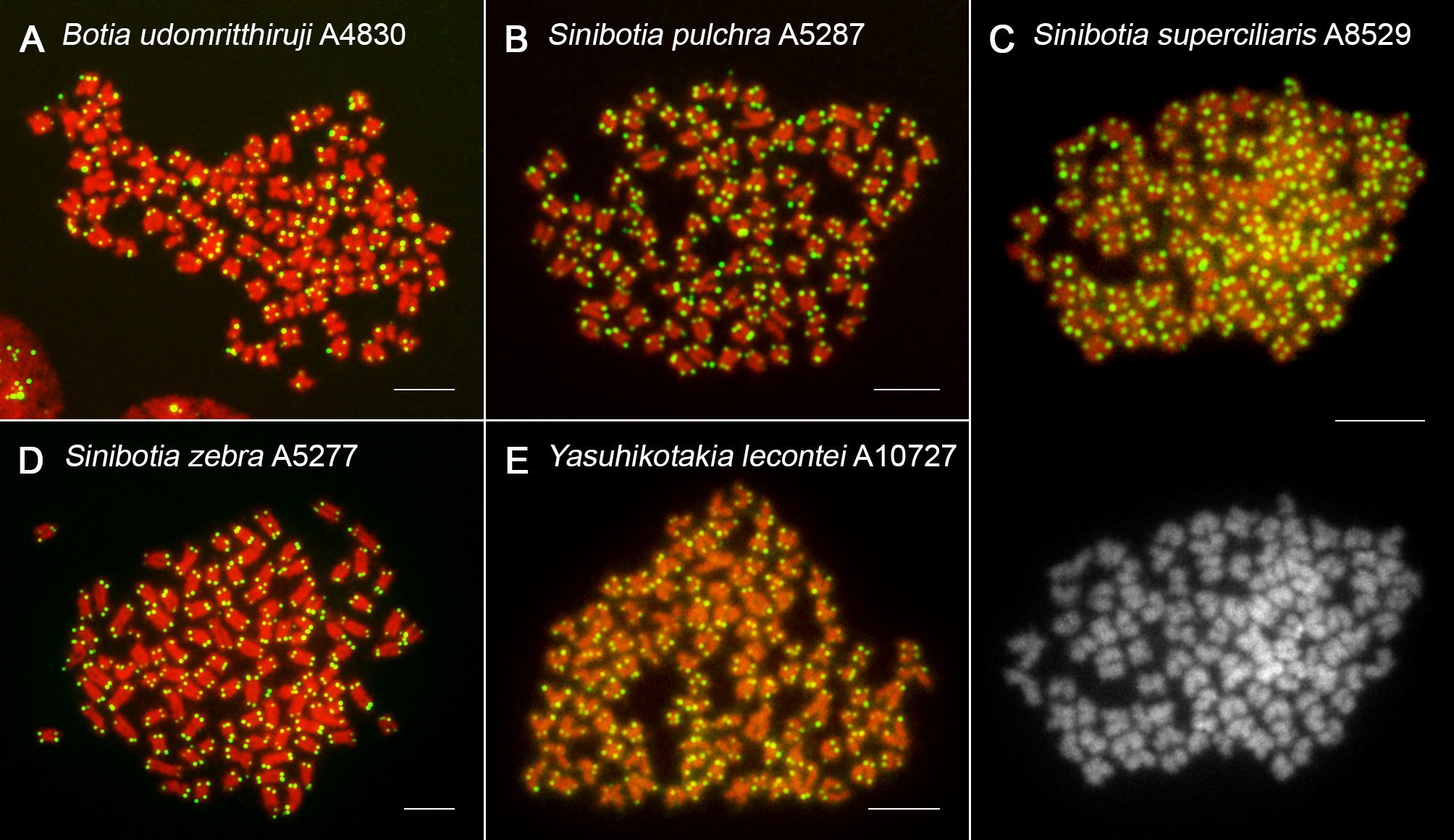

Supplement: S6 Fig — (A) B. udomritthiruji, (B) S. pulchra, (C) S. superciliaris, (D) S. zebra, (E) Y. lecontei. For better contrast, pictures were pseudocoloured in green (telomeric repeat probe) and red (DAPI). For better distinction between individual chromosomes, we included also the separate image with DAPI channel for S. superciliaris (C). Bar = 10 μm. (TIF) [file pone.0195054.s006.tif]
